# Effect of melatonin on electrical impedance and biomarkers of damage in a gastric ischemia/reperfusion model

**DOI:** 10.1371/journal.pone.0273099

**Published:** 2022-08-16

**Authors:** Eduardo Peña-Mercado, Mario Garcia-Lorenzana, Sara Huerta-Yepez, Anahis Cruz-Ledesma, Nohra E. Beltran-Vargas

**Affiliations:** 1 Departamento de Procesos y Tecnologia, Universidad Autonoma Metropolitana, Unidad Cuajimalpa, CDMX, Mexico; 2 Departamento de Biologia de la Reproduccion, Universidad Autonoma Metropolitana, Unidad Iztapalapa, CDMX, Mexico; 3 Unidad de Investigacion en Enfermedades Hematooncologicas, Hospital Infantil de Mexico, Federico Gomez, CDMX, Mexico; 4 Universidad Autonoma Metropolitana, Unidad Xochimilco, CDMX, Mexico; City of Hope National Medical Center, UNITED STATES

## Abstract

The damage to the gastrointestinal mucosa induced by ischemia/reperfusion (I/R) is closely related to high mortality in critically ill patients, which is attributable, in part, to the lack of an early method of diagnosis to show the degree of ischemia-induced injury in this type of patients. Electrical Impedance Spectroscopy (EIS) has been shown to be a tool to early diagnose gastric mucosal damage induced by ischemia. A therapeutic alternative to reduce this type of injury is melatonin (MT), which has gastroprotective effects in I/R models. In this work, the effect of treatment with MT on the electrical properties of gastric tissue, biomarkers of inflammatory (iNOS and COX-2), proliferation, and apoptotic process under I/R conditions in male Wistar rats was evaluated through EIS, histological and immunohistochemical analysis. Treatment with MT prevents gastric mucosa damage, causing a decrease in gastric impedance parameters related to the inflammatory process and cellular damage. This suggests that EIS could be used as a tool to diagnose and monitor the evolution of gastric mucosal injury, as well as in the recovery process in critically ill patients.

## Introduction

Gastrointestinal I/R injury is considered to be the initial factor of systemic inflammatory response syndrome and multiple organic dysfunction syndrome, which results in high mortality in clinical practice. This type of injury can cause dysfunction of the epithelial barrier and at the same time cause serious damage to the mucosa, which, in addition to fragile immunization presented in critically ill patients, are the main cause of serious complications and death [1, 2]. This type of injury is associated with high mortality rates ranging from 60 to 90%, which have not changed in the last 70 years. High mortality is attributable, in part, to the lack of predictive diagnostic testing that allows early detection in patients with high risk of gastrointestinal ischemia [3]. In critically ill patients, markers of splanchnic ischemia such as serum lactate and white blood cell count have been shown to lack sensitivity and specificity, while radiological diagnostic tests expose patients to the risk of nephropathy and are also not definitive [3, 4].

Due to difficulties in early diagnosing gastrointestinal ischemia, the use of electrical impedance spectroscopy (EIS) has been proposed, which measures frequency-dependent opposition to the flow of electric current and has resistive and capacitive characteristics. The strongly ionic intra and extracellular environment contributes to the resistive component of response (resistance (R)), while cell membranes contribute to capacitive effects (reactance (X)) [5]. In ischemic conditions, there are changes in extra and intracellular volume, in addition to alterations in the cell the measurements in R and X, respectively; therefore, changes in R are associated with inflammation while damage to cell membranes is related to changes in X. Our team has used EIS to monitor and detect the degree of gastric mucosa injury associated with ischemic conditions in critically ill patients and in murine models [6, 7]. In addition, a Tissue Lesion Index (TLI) has been proposed, in which the quantification of histopathological alterations was considered, and a score was assigned according to the scales presented in Table 1. A score >3 suggests that tissue damage exists [8]. Based on the relationship between changes in gastric impedance parameters (Resistance and Reactance) and the TLI score, Peña-Mercado *et al*. [9] proposed thresholds for Low Resistance (R_L_) frequencies and High Reactance (X_H_) frequencies to identify reversible (sublethal) lesions in the gastric mucosa (cell edema and inflammatory process) and irreversible (loss of glandular architecture). From these, reversible lesions were identified at 60 min of ischemia, while at 90 min of ischemia there are acute gastric lesions (hemorrhage, erosion) that compromise the integrity of the gastric mucosa.

**Table 1:** Scale to indicate the score for each variable.

score	0	1	2
**Vascular area (μm** ^**2**^ **)**	100–300 μm^2^	301–600 μm^2^	≥601 μm^2^
**Glandular lumen area**	50–300 μm^2^	301–300 μm^2^	≥601 μm^2^
**Celular injury (number of cells with edema, piknosis or karyolysis)**	≤25	26–85	≥86
**Epithelial erosion**	Whitout erosion	Superficial epithelial erosion	Erosion to the oxyntic portion

Diverse factors are related to the pathogenesis of ischemia/reperfusion (I/R) induced damage, including dysfunction in energy metabolism, oxidative and nitrosative stress, dysfunction of antioxidant enzymes, calcium overload, apoptosis, and production of inflammatory mediators (cytokines, prostaglandins, reactive oxygen species (ROS) and nitrogen) [10]. Inducible nitric oxide synthase (iNOS) is an enzyme related to the inflammatory process and overproduction of nitric oxide (NO), which has been shown to be present in tissue damage under reperfusion conditions [11]. Whereas cyclooxygenase-2 (COX-2) is an enzyme that has been identified as a marker of inflammatory process, as its induction is regulated by inflammatory agents such as endotoxins and cytokines, and its increase in gastrointestinal I/R conditions has been also identified [12, 13].

Gastrointestinal I/R injury is always followed by the proliferation and differentiation of epithelial cells to properly reconstruct the epithelium; however, insufficient proliferation and regeneration to fully rescue gastrointestinal mucosal barrier function can be seen by the high mortality rate in clinical practice [1].

Melatonin (MT) (N-acetyl 5-methoxytryptamine) is a hormone secreted by the pineal gland which has a broad regulatory role. Diverse physiological functions of MT have been demonstrated, including the regulation of the circadian and endocrine rhythm, and its anti-inflammatory, analgesic, anxiolytic and antioxidant activity [14]. MT has been detected in different extra-pineal tissues including brain, choroid plexus, retina, lens, cochlea, gastrointestinal tract, skin, ovaries, lymphocytes, macrophages, and endothelial cells [15, 16]. In the gastrointestinal tract, MT is produced in enterochromaffin cells and is independent of the circadian cycle [16]. It regulates the transport of ions and water, epithelial proliferation, acid secretion and motility [17].

In preclinical studies, it has been shown that both pretreatment (preventive) and treatment (corrective) with MT has gastroprotective effects after inducing acute lesions in the gastric mucosa by I/R. Due to its antioxidant properties, it reduces oxidative stress, increases the activation of antioxidant enzymes and the production of prostaglandins, and decreases neutrophil infiltration and gastric mucosal cell apoptosis [18–20]. However, the effect of treatment with MT on the electrical properties of the gastric mucosa after inducing I/R lesions has not been studied to date.

The objective of this work was to evaluate the effect of MT treatment on gastric impedance parameters and relate them to inflammatory process biomarkers and apoptosis, after inducing sublethal lesions by ischemia and 24 hours of reperfusion.

The administration of MT induced a preventive effect on the gastric mucosa damage reducing the measurements of gastric impedance parameters, the expression of biomarkers of the inflammatory process, in addition to decreasing apoptosis and increasing proliferation.

## Materials and methods

### Ethical considerations and preparation of animals

The experiments in rats were conducted in accordance with the guidelines and approval of the Ethics Committee of the National Center for Research in Medical Imaging and Instrumentation (Protocol number: EBD_AC_03_17) under the Mexican Official Standard (NOM-062-ZOO-1999). Twenty-four male Wistar rats (250–300 g) were used provided by the vivarium of the Universidad Autonoma Metropolitana–Iztapalapa. The animals were housed under a controlled environment (22±2°C; 50–60% relative humidity and 12h-12h light-dark cycles) with access to food and water *ad libitum* until the time of surgery. At the end of the experiments, the rats were sacrificed with an overdose of pentobarbital (NOM-062-ZOO-1999).

### Surgical procedure

Induction of anesthesia was done through intraperitoneal (i.p) administration of a mixture of xylazine and ketamine (10–90 mg/kg) and were maintained with 2% inhaled isoflurane during the procedure. Rats were randomly assigned in 3 groups (n = 8 per group): Sham, Ischemia/Reperfusion (I/R) and Ischemia/Reperfusion + Melatonin (I/R+MT).

For the I/R group, the gastric mucosal lesion was caused by the model proposed by Wada *et al*. [21], the celiac artery was occluded to cause ischemia for 60 min with hemostatic forceps; the tip was lined with parafilm to prevent injury to the blood vessel. The occlusion was confirmed with the change in color of the gastric tissue, which returned to its normal color in the reperfusion period. Five minutes before removal of the occlusion, 300 μL of the vehicle (ethanol 1%) was administered and was subsequently released to allow reperfusion for 24 hours. In the I/R+MT group, 10 mg/kg MT (SC-207848, Santa Cruz Biotechnology) was administered via i.p 5 min before allowing 24 hours of reperfusion. MT was dissolved in 300 μL of ethanol 1%, diluted with 0.9% saline [19]. In the Sham group, the surgical procedure was performed without inducing ischemia. During the surgical procedure, the temperature of the animals remained controlled with a thermostatic electric blanket heated to 37°C. After 24 hours of reperfusion, measurements of impedance spectra and glandular portion biopsies were taken for histological and immunohistochemical analysis.

### Gastric impedance measurements

The gastric impedance spectra were obtained by means of a spectrometric nasogastric probe (SNG/E), which contains four silver electrodes at its distal end. An alternating electric current (1 mA) was applied to the mucosa through the external electrodes and the spectra were recorded at 23 frequencies ranging from 215 Hz to 1MHz. The device measured the tissue voltage and calculated impedance parameters (Resistance and Reactance). In the gastric mucosa, spectroscopy allows to identify two areas of dispersion specific to this tissue, one zone at frequencies less than 10 kHz (low frequencies) and the other above 10 kHz (high frequencies). The spectra obtained during the experiments were processed offline using a mathematical model to calculate the characteristic parameters of the gastric mucosa: Resistance (R_L_) at Low frequencies and Reactance (X_H_) at High frequencies.

### Histological and immunohistochemical analysis

For histological analysis, biopsies of the glandular portion were taken and fixed with stabilized neutral formalin (10%). Biopsies were dehydrated to varying degrees of ethyl alcohol, diaphanized with xylene, and embedded in paraffin within a tissue microarray. For morpho-pathological analysis, transverse and longitudinal cuts with a thickness of 3 μm were obtained and subsequently stained with Hematoxylin-Eosin (H-E). The severity of the gastric mucosal lesion was quantified by a proposed Tissue Lesion Index (TLI) [8]. To evaluate the production of mucins, longitudinal cuts were made 3 μm thick, which were stained with Periodic Acid-Schiff -Alcian Blue (PAS-AB).

For immunohistochemical analysis, the slices were deparaffined and the recovery of antigens was done with sodium citrate buffer (pH 6). Endogenous peroxidase was blocked with hydrogen peroxide (3%) for 30 min and the blocking of non-specific bindings was performed for 3 hours. Immunodetection was done with the following primary antibodies: anti-iNOS (1:100, Rabbit polyclonal, Santa Cruz Biotechnology, SC-649), anti-COX-2 (1:1000, Rabbit polyclonal, Novus, NB-100-689) and anti-PCNA (1:1000, Rabbit polyclonal, ABCAM, AB-2426) and incubation took place overnight at 4°C. An Isotype control was performed as a negative control (CalbiochEM millipore, NI01-100 (rabbit); NI02-100 (goat)). Gastric mucosa biopsies subjected to 90 min of ischemia were used for the positive control. The secondary antibody conjugated with horseradish peroxidase (HRP) (antirabbit: MP-7401, antigoat: MP-7405; Vector Laboratories) was incubated for 30 min. The antigen-antibody complex was revealed with an immunodetection kit (Vector Laboratories) and the counterstain was done with hematoxylin. Histological (H-E) and quantitative immunohistochemical analysis was performed through digital pathology. The slices were scanned and digitized (Aperio CS2, Leica Biosystems) to quantify the intensity of staining (Intensity/area) in immunostained slices for iNOS and COX-2, as well as the number of PCNA-positive nuclei in the gastric mucosa (3 fields per sample, ×100).

### Apoptosis assay (TUNEL)

To detect DNA fragmentation, an apoptosis assay TUNEL (Terminal dUTP nick-end labeling) was performed with the *In situ* cell death detection fluorescein kit (Roche Diagnostics, Indianapolis, USA). Longitudinal cuts 5 μm thick were obtained, deparaffined and the antigens were recovered with sodium citrate buffer (20× Immuno/DNA Retriever with Citrate, Santa Barbara, California, USA); they were incubated with the fluorescein conjugated TUNEL reaction mixture for 60 min at room temperature. The slices were mounted with Fluoroshield^TM^ with DAPI (Sigma-Aldrich, St. Louis, USA). For quantitative analysis, three areas of the upper portion of the gastric mucosa (×400) per cut were obtained and the number of TUNEL-positive nuclei was counted. The slices were observed with the Axiovert 40 CFL (Carl Zeiss) inverted microscope, the images were digitized and analyzed with ZEN software (Carl Zeiss).

### Statistical analysis

Differences in impedance parameters between the groups was analyzed with an ANOVA test followed by a *post hoc* Tukey test. To analyze changes in TLI score, expression of inflammatory process biomarkers, apoptosis, and proliferation, a Kruskal Wallis test was performed followed by a *post hoc* T3 Dunnett test. The data are presented as the mean ± SEM, and the median and the interquartile range (IQR). The value of *p* < 0.01 was considered to indicate statistically significant differences. Statistical analysis was performed with SPSS v21.0 software (IBM Corp, Armonk, NY, USA) and GraphPad Prism 9 (GraphPad Software, San Diego, CA, USA).

## Results

### Effect of MT on changes in gastric impedance parameters induced by I/R

**Fig 1:**
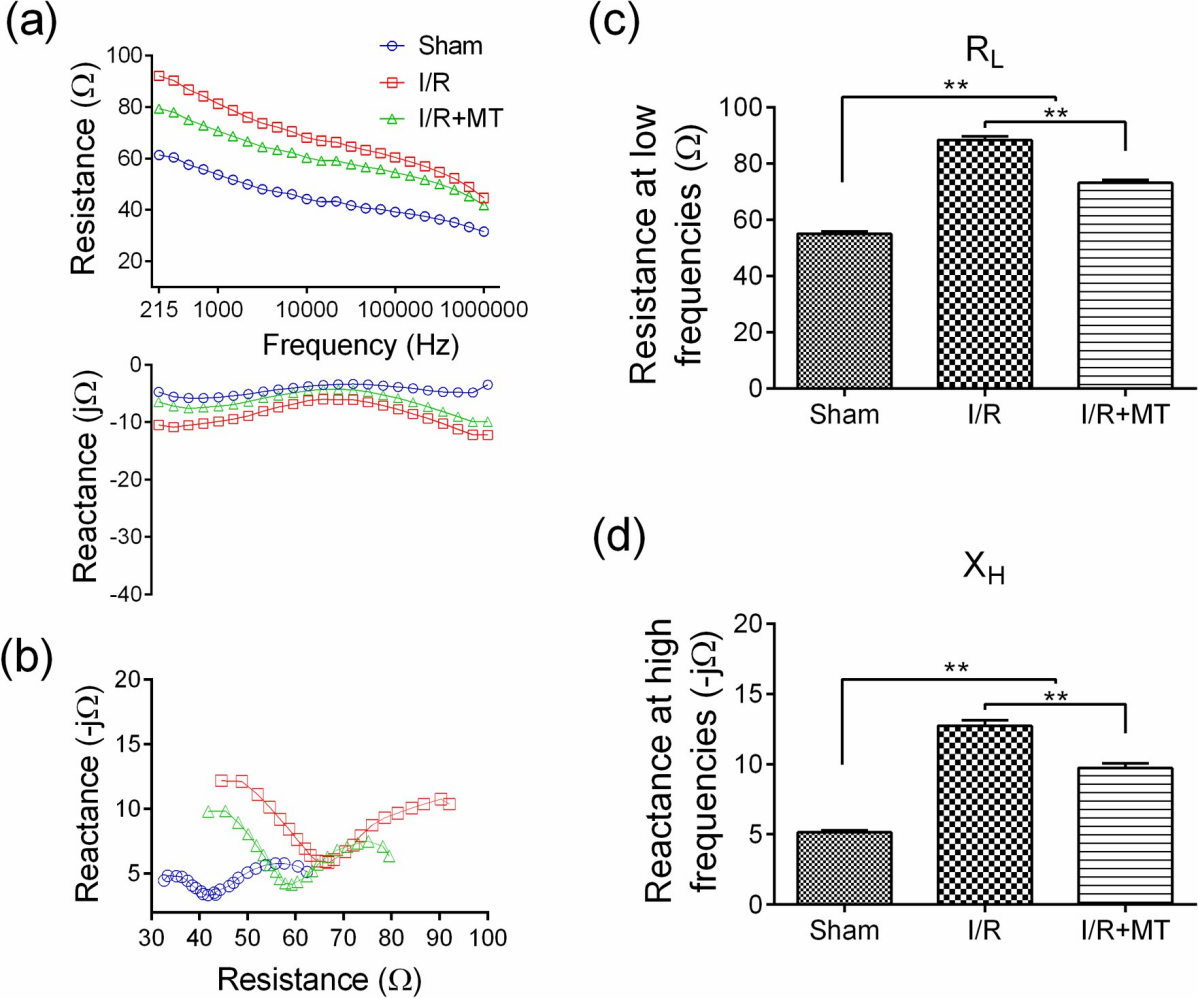
Effect of MT on the gastric mucosa impedance in experimental groups. (a) Graphic representation of the gastric impedance spectra (Resistance and Reactance) of the experimental groups: Sham, I/R and I/R+MT. (b) Cole-Cole plot (Resistance vs Reactance). Graphic representation of impedance parameters of the experimental groups: (c) Resistance at Low frequencies (R_L_) and (d) Reactance at High frequencies (X_H_). An ANOVA statistical test was performed followed by a Tukey *post hoc* test. The results are presented as the mean ± SEM. ** *p* < 0.001. Treatment with MT reduces measurements of gastric impedance parameters after the I/R model.

To assess the effect of MT on changes in the electrical properties of the gastric mucosa subjected to I/R, the impedance spectra of the different groups were recorded (Fig 1A); additionally, Resistance vs Reactance (Cole-Cole plot) was graphed (Fig 1B). Fig 1A showed an increase in Resistance and Reactance at Low frequencies (< 10000 Hz) in the I/R group with respect to the Sham group, which is significantly reduced in the I/R+MT group. Similarly, at High frequencies (> 10000 Hz), both parameters (Resistance and Reactance) increased after reperfusion and decreased with treatment. On the other hand, the Cole-Cole plot (Fig 1B) showed a significant shift to the right side in the I/R group which decreased in the treated group, suggesting that there is a possible reduction of the damage after the administration of MT.

Measurements of R_L_ and X_H_ (Fig 1C and 1D) showed a statistically significant increase (*p* < 0.001) in the I/R and I/R+MT groups with respect to the Sham group, with the I/R group having the largest increase, which decreased in the I/R+MT group.

### Effect of MT on gastric mucosal damage induced by I/R

**Fig 2:**
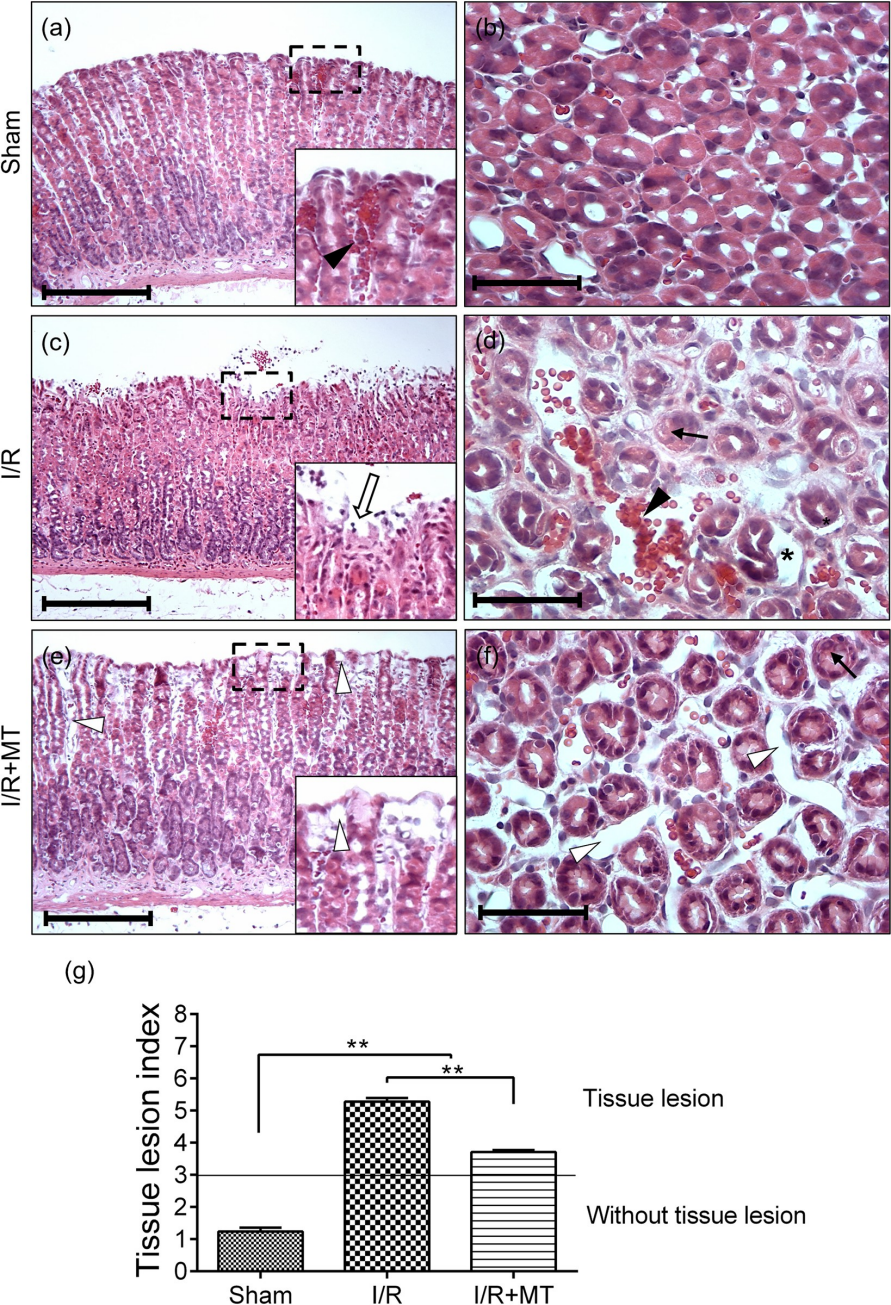
Effect of MT on histopathological alterations in the experimental groups. Representative photomicrographs of longitudinal (a), (c) and (e) and transverse (b), (d) and (f) sections of rat gastric mucosa. Sham (a) and (b), I/R (c) and (d) and I/R+MT (e) and (f) groups. Framed regions were enlarged to show (a) vascular congestion, (b) epithelial erosion and (c) granulation tissue. Identifiers: (black arrowhead) vascular congestion, (white arrow) epithelial erosion, (black arrow) damaged cell, (⁎) epithelial detachment, (white arrowhead) vascular dilation. H-E. Scale bar: 200 μm (a), (c) and (e). Scale bar: 100 μm (b), (d) and (f). (g) Graphic representation of the TLI score. A Kruskal Wallis statistical test was performed followed by a Dunnett *post hoc* T3. The results are presented as the mean ± SEM. ** *p* < 0.001. Administration of MT accelerated the re-epithelization process in the gastric mucosa after 60 min of ischemia and 24 hours of reperfusion.

Histopathological analysis in the Sham group (Fig 2A and 2B) showed some areas of vascular congestion in the upper portion of the gastric mucosa; however, the mucosal architecture is preserved. In the I/R group (Fig 2C and 2D), congested blood vessels and hemorrhagic areas were observed, as well as exfoliation of the superficial epithelium and some areas of erosion. In the I/R+MT group (Fig 2E and 2F), histological re-epithelization data were observed, superficial and glandular epithelium cells showed cytoplasmic changes prone to basophilia. In the magnification (Fig 2E), flattened epithelial cells were observed lining the blood vessels and the lamina propria. In addition, slight interstitial edema was identified with extensive presence of dilated blood vessels without the presence of erythrocytes mainly in the upper part of the mucosa (Fig 2F). These histological data correspond to granulation tissue mainly in the upper portion of the gastric mucosa.

Quantitative analysis showed a statistically significant increase (*p* < 0.001) in the TLI score in the I/R and I/R+MT groups with respect to the Sham group, with the I/R group having the greatest increase, which decreases with the treatment of MT. In both groups a score > 3 was obtained indicating that there are tissue alterations. However, in the I/R group, the damage to the gastric mucosa is more severe (Fig 2G).

**Fig 3:**
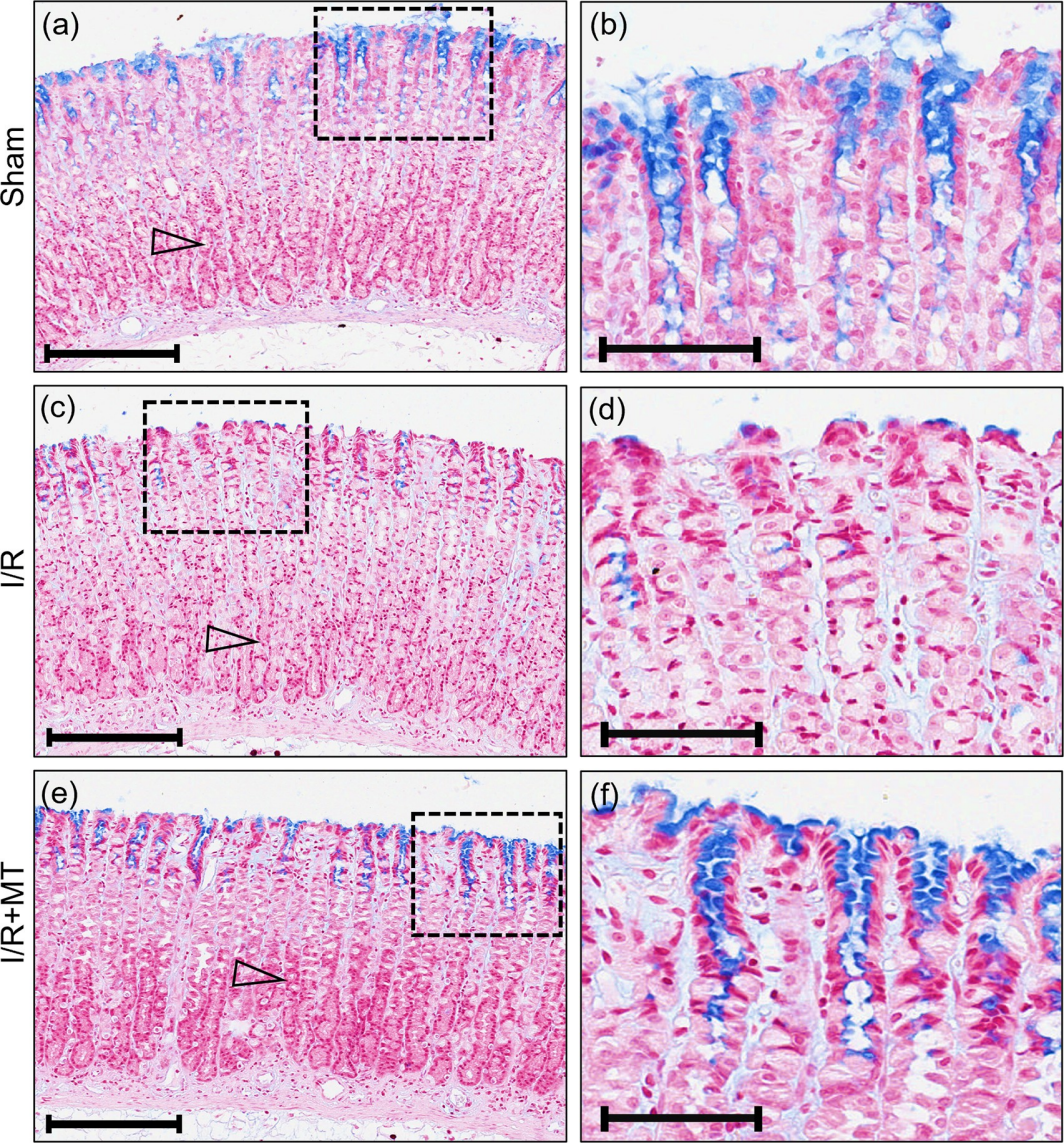
Effect of MT on alterations in mucinogen secretion in experimental groups. Representative photomicrographs of longitudinal sections (a) to (f) of rat gastric mucosa: Sham (a) and (b), I/R (c) and (d) and I/R+MT (e) and (f) groups. Identifier: (hollow arrowhead) PAS-positive cells. Scale bar: 200 μm (a), (c) and (e). Magnification: Scale bar: 100 μm (b), (d) and (f). Framed regions were enlarged to show the increase in mucin production in the superficial epithelium after MT treatment in the I/R model.

PAS-AB staining identified the location and density of AB-positive mucus-producing cells (blue color) and PAS-positive (bright magenta color), which produce acidic and neutral glycosaminoglycans, respectively. Alcianophilic mucus-producing cells (blue color) were identified in the surface epithelium of the gastric mucosa in the Sham group (Fig 3A and 3B), while in the I/R group (Fig 3C and 3D) a decrease in tinctorial affinity was observed in the surface epithelium and foveola areas, which increased after treatment with MT (Fig 3E and 3F) compared to the reperfusion group. In addition, PAS-positive cells were found located mainly in the upper and fundic region of the gastric mucosa in the Sham group (Fig 3A). The staining affinity in PAS-positive cells decrease after I/R (Fig 3C) and increased with treatment administration (Fig 3E).

To identify the effect of MT on the degree of injury induced by I/R, a scatter plot was made with the TLI score and gastric impedance parameter thresholds, R_L_ (no damage: <70 Ω, reversible damage: 70 to 87 Ω, irreversible damage: >87 Ω) and X_H_ (no damage: < -7.7 Ω, reversible damage: -7.7 to -13.5 Ω, irreversible damage: > -13.5 Ω) that were reported by Peña-Mercado *et al*. [9], which allow to identify reversible gastric mucosal lesions (cell edema and inflammatory process) and irreversible (loss of glandular architecture).

**Fig 4:**
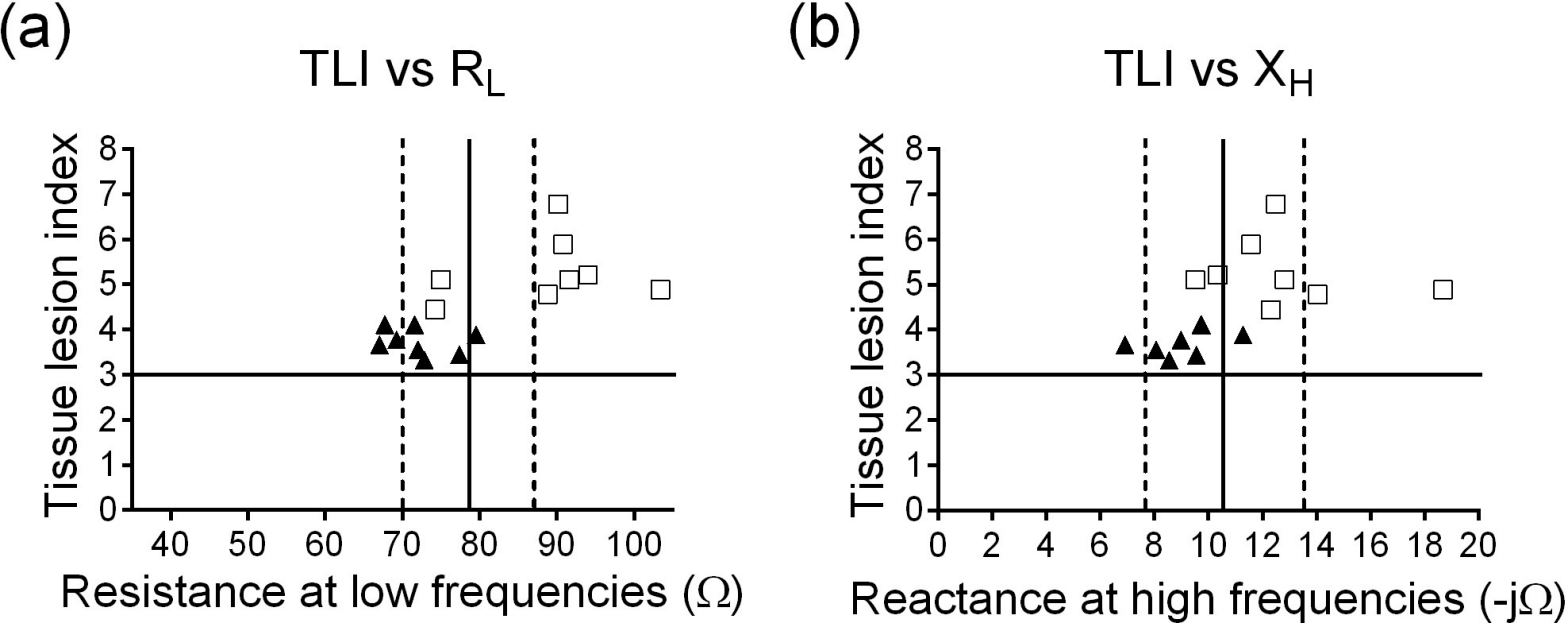
Comparison of the TLI score vs the impedance parameters (R_L_ and X_H_). Identifiers: (□) I/R group, (▲) I/R+MT group.

Fig 4 shows the TLI score, in which it is observed that it is larger in the I/R group than in the I/R+MT group. With respect to R_L_ and X_H_, it was identified that the data of the group treated with MT are within the reversible damage zone. While in the I/R group, some data are in the irreversible damage zone. Taken together, it can be inferred that MT treatment prevents from gastric mucosal damage induced by I/R.

### Effect of I/R treatment with MT on the expression of iNOS, COX-2, PCNA and apoptosis

**Fig 5:**
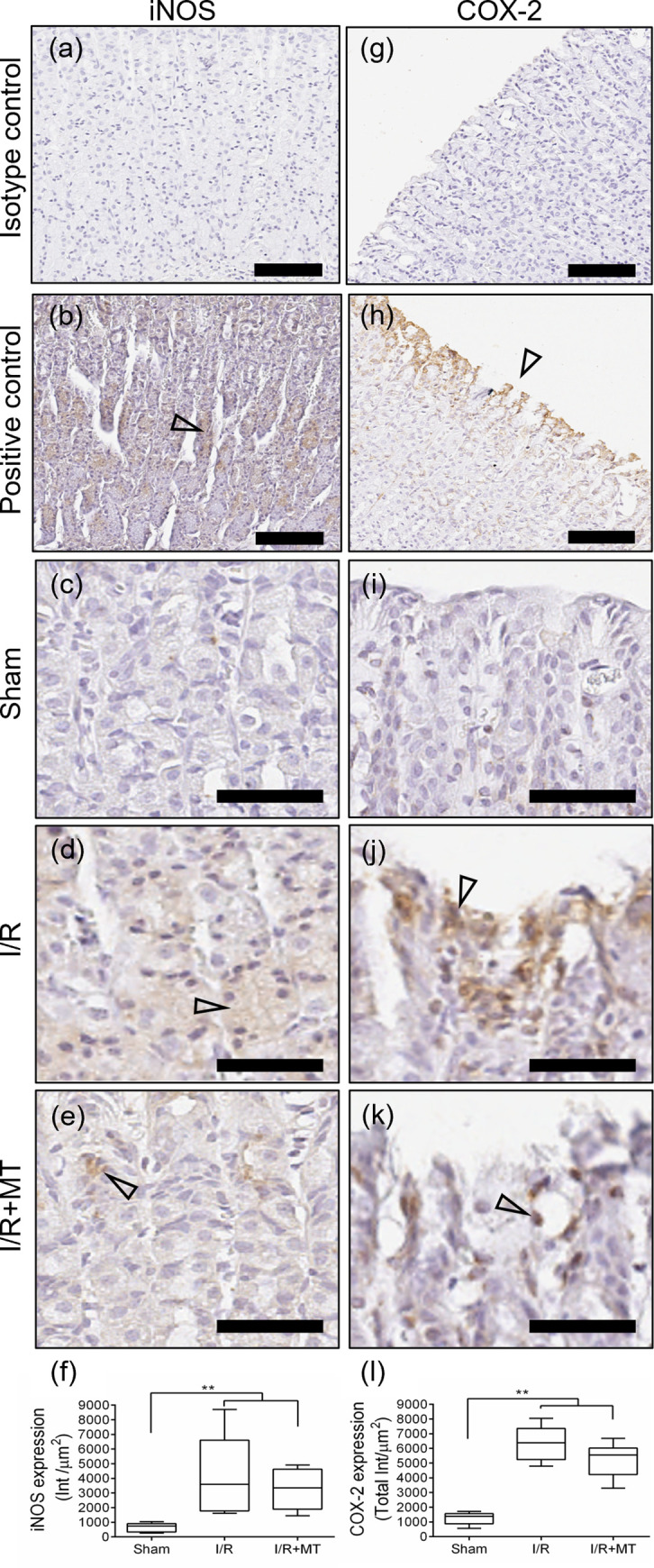
Effect of MT on inflammatory process markers in experimental groups. Representative photomicrographs of the Isotype control (a) and (g), Positive control (b) and (h), immunodetection of iNOS (c) to (e) and COX-2 (i) to (k). Identifier: (hollow arrowhead) cells with positive immunoreactivity. Scale bar: 200 μm (a), (b), (g) and (h). Scale bar: 100 μm (c) to (e) and (i) to (k). Graphic representation of the quantification of the expression of iNOS (f) and COX-2 (l). A Kruskal Wallis statistical test was performed followed by a Dunnett *post hoc* T3. The results are presented as the median and the IQR. ** *p* < 0.001. The expression of markers of inflammatory process decreased with treatment with MT after induction of lesions in the gastric mucosa by I /R.

To evaluate the effect of MT on biomarkers of inflammatory process, the expression of iNOS and COX-2 in the gastric mucosa was determined. Immunohistochemical analysis of iNOS (Fig 5A–5E) showed cytoplasmic positive immunoreactivity in gastric mucosal epithelium cells in I/R and I/R+MT groups, with the I/R group being the one with the largest increase over the Sham group (*p* < 0.001), this increase tends to decrease after treatment with MT (Fig 5F). Immunodetection of COX-2 (Fig 5G–5K) showed positive immunoreactivity under reperfusion conditions, COX-2 expression was observed in endothelial cells, immune system cells, in addition to identifying a significant level of expression in zones of gastric mucosa exfoliation and erosion (Fig 5J). After treatment with MT, expression was found mainly in immune system cells and some epithelial exfoliation areas (Fig 5K). Quantitative analysis showed a statistically significant increase (*p* < 0.001) in COX-2 expression with respect to the Sham group, while in the MT-treated group, a decrease was identified with respect to the reperfusion group (Fig 5L).

**Fig 6:**
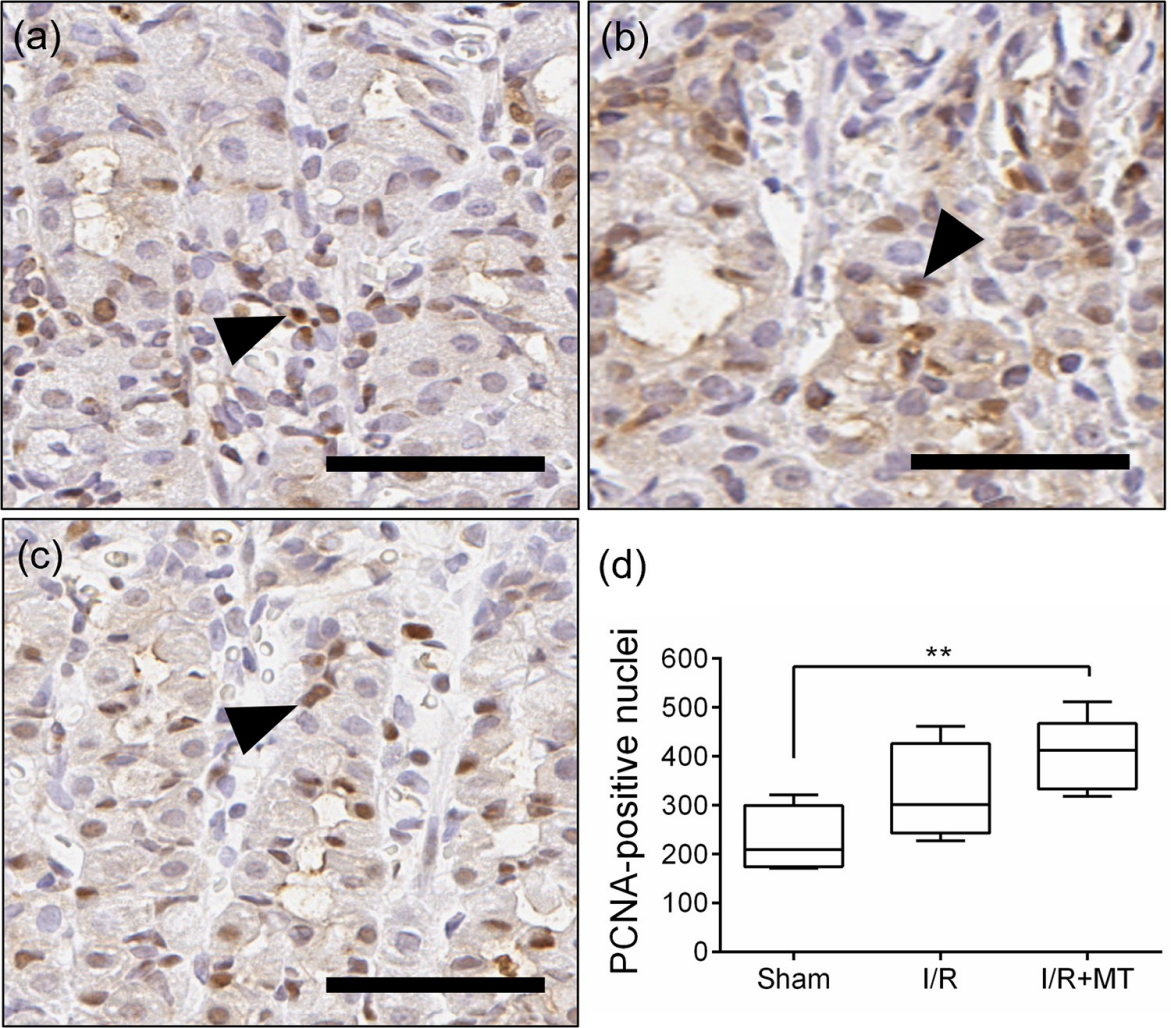
Effect of MT on proliferation in experimental groups. Representative photomicrographs of PCNA immunodetection (a) to (c). Identifiers: (black arrowhead) PCNA-positive nuclei. Scale bar: 100 μm. Graphic representation of the quantification of the number of PCNA-positive (d). A Kruskal Wallis statistical test was performed followed by a Dunnett *post hoc* T3. The results are presented as the median and the IQR. ** *p* < 0.001. Treatment with MT increased proliferation in glandular epithelium cells in the I/R model.

To evaluate the effect of MT on cell proliferation in the gastric mucosa under I/R conditions, PCNA immunohistochemical marking was performed (Fig 6A–6C). Some PCNA-positive nuclei were found in some cells located in the lamina propria and foveolar epithelium, as well as in the portion of the neck and the bottom of the gland (Fig 6A) in the Sham group. While in the I/R group, PCNA-positive nuclei were observed in epithelial cells in the portion of the isthmus and neck of the glands, mainly in erosion zones (Fig 6B). On the other hand, the I/R+MT group identified positive immunoreactivity in cell nuclei located in the lamina propria, epithelial cells of the isthmus and the portion of the neck, and some in the foveolas (Fig 6C). Quantitative analysis showed an increase in the number of positive nuclei in the treated group with respect to the Sham (*p* < 0.001) and I/R groups (Fig 6D).

**Fig 7:**
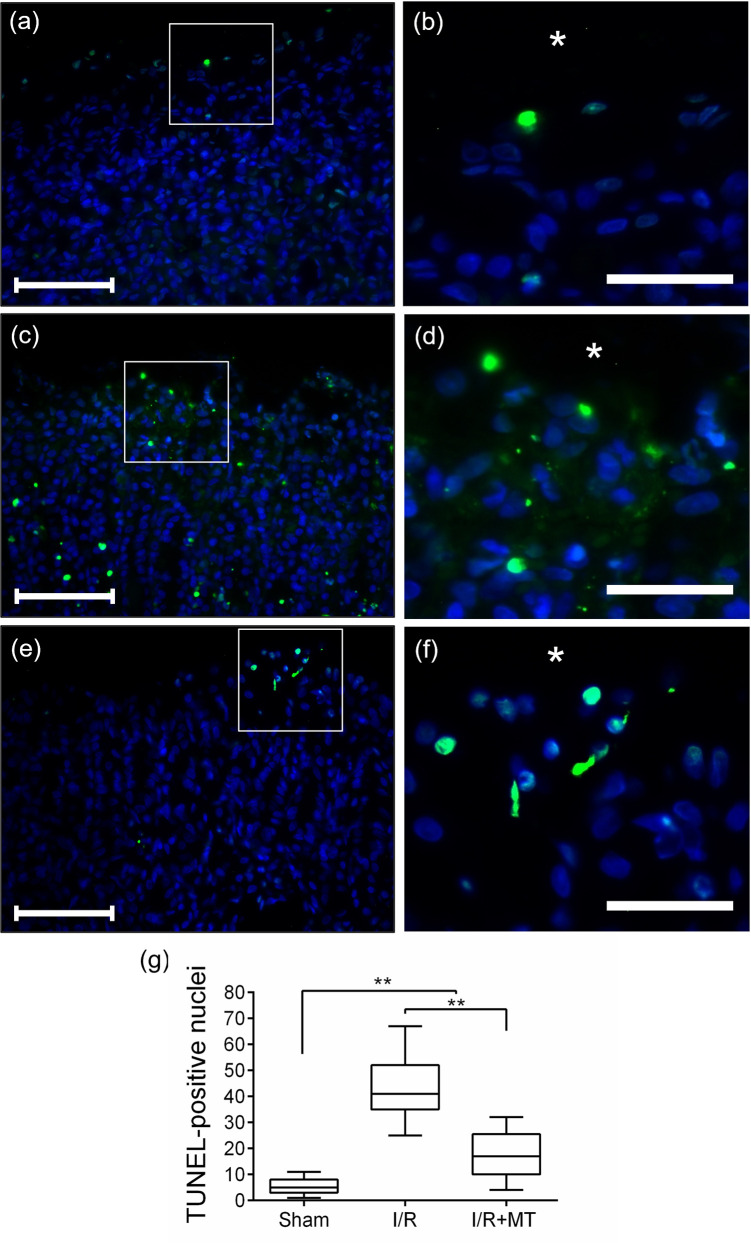
Effect of MT on apoptosis in experimental groups. Representative photomicrographs of TUNEL (Terminal dUTP Nick-End Labeling) apoptosis assay (a) to (f). Identifiers: (Framed area) TUNEL-positive cells, (⁎) gastric lumen. (a). Scale bar: 100 μm (a), (c) and (e). Magnification: Scale bar: 50 μm (b), (d) and (f). Graphic representation of the quantification of the TUNEL-positive nuclei (g). A Kruskal Wallis statistical test was performed followed by a Dunnett *post hoc* T3. The results are presented as the median and the IQR. ** *p* < 0.001. Treatment with MT decreased apoptosis in glandular epithelium cells in the I/R model.

To examine the effect of MT on apoptosis induced by I/R, an apoptosis assay TUNEL was performed. The Sham group (Fig 7A and 7B) showed some apoptotic cells in the surface epithelium of the gastric mucosa. Under reperfusion conditions (Fig 7C and 7D), a greater number of apoptotic cells were identified in both the surface epithelium and the oxyntic portion of the mucosa, while in the MT-treated group (Fig 7E and 7F), apoptotic cells were found in lower quantity than in the I/R group, mainly confined to the upper portion of the mucosa, this suggests that the proliferation process has displaced the apoptotic cells into the gastric lumen. In the quantitative analysis, a statistically significant increase (*p* < 0.001) was observed in the number of TUNEL-positive cells in the I/R group with respect to Sham, which decreased in the I/R+MT group (Fig 7G). With this result, it was observed that treatment with MT attenuates apoptosis in the gastric mucosa induced by I/R.

Together, these results suggest that administration of MT after induction of ischemic sublethal lesions in the gastric mucosa may reduce the degree of tissue injury, due to its anti-inflammatory and anti-apoptotic properties (Table 2).

**Table 2:** Effect of MT treatment on impedance parameters (R_L_ and X_H_), gastric mucosa damage and the expression of inflammatory process markers, proliferation, and apoptosis.

	Sham	I/R	I/R+MT
**Impedance parameters**			
R_L_ (Ω)	52.57 ± 0.70	88.49 ± 1.23	72.17 ± 0.94
X_H_ (-jΩ)	5.17 ± 0.18	12.72 ± 0.40	9.10 ± 0.31
**Histology**			
TLI	1.23 ± 0.11	5.27 ± 0.11	3.73 ± 0.08
**Immunohistochemistry**			
iNOS expression (Int/μm^2^)	744.9 (410.2–911.2)	3589 (1963–6357)	3350 (2122–4421)
COX-2 expression (Int/μm^2^)	1392 (1054–1444)	6383 (5716–6945)	5556 (4661–5733)
PCNA-positive nuclei	209.2 (169.5–282.9)	301.1 (261.6–384.4)	412.2 (356–459.1)
**Immunofluoresce**			
TUNEL-positive nuclei	5 (3.8–6.7)	41 (35.8–50.4)	17 (14.42–22.15)

The results are presented as the mean ± SEM (R_L_, X_H_ and TLI) and the median and the IQR (iNOS, COX-2, PCNA and TUNEL).

## Discussion

Gastrointestinal dysfunction is frequently observed in critically ill patients, generally referring to functional deterioration of the gastrointestinal tract, which can lead to multiple organ failure [22]. Recently, it has been proposed that the degree of severity of acute gastrointestinal injury is a good predictor of mortality in this type of patients [23], which is why early diagnosis is essential to usher possible therapeutic interventions to avoid complications.

The EIS has the ability to show morphological alterations in tissue under I/R conditions in different neoplastic organs and processes[24–26]. Additionally, it has been shown to have the sensibility to detect cellular changes related to the processes of proliferation, re-epithelization, regeneration, and healing in both *in vitro* and *in vivo* models [27–30].

The results of the qualitative and quantitative histological analysis in our I/R model showed lesions in the gastric mucosa (edema, hemorrhage, and some areas of erosion in the foveolar portion) that could compromise the barrier function. In relation to these results, Cabeza *et al*. [31], reported hemorrhagic, edematous lesions, as well as areas of exfoliation and structural alterations in 2/3 of the parts of the glandular pits. Recently, Mubarak *et al*. [20], reported edema and disruption of the architecture of the gastric mucosa with marked necrosis and apoptosis of the fundic glands, as well as infiltration of inflammatory cells throughout the thickness of the mucosa. The injuries described by both authors are apparently more severe than those observed in this work, this could be because their analysis was done on acute lesions induced by 60 min of reperfusion, while in our model, the time of reperfusion is 24 hours and there is already a process of recovery of the gastric mucosa [32]. On the other hand, in the I/R+MT group, morphological features of the regeneration of the gastric mucosa were observed as re-epithelization and vascular granulation tissue in the luminal portion of the gastric mucosa after treatment with MT. Granulation tissue develops in areas of injury and consists of connective tissue cells in proliferation (macrophages and fibroblasts) and endothelial cells by which capillaries form through the neovascularization process, which is stimulated by angiogenic peptides such as vascular endothelial growth factor [33]. Morphological results associated with the restitution process in a model of gastric injury induced by ethanol exposure have been reported by Ito & Lacy [34]. They identified epithelial discontinuities located on capillaries, the superficial capillaries contained few erythrocytes and appeared dilated by plasma. Some restored areas had columnar cells of almost normal appearance, but many regions had low or very thin cubic cells spread over the basal lamina. The findings presented in this paper reinforce previous studies that report that MT treatment plays a restorative role after the I/R-induced injury [20].

These results identified histopathological alterations that induce changes in gastric impedance parameters. The increase in R_L_ is related to cell edema and alterations in extracellular volume [29]. Under reperfusion conditions, epithelial cells can be found in the process of edema. At the level of the lamina propria there is a generation of vascular congestion and zones of hemorrhage (abundant presence of erythrocytes) as well as a presence of inflammatory cells (macrophages, neutrophils) [20, 31], these tissue characteristics reduce extracellular space causing the increase of R_L_, while the increase in X_H_ is related to the loss of membrane integrity and cell death [35]. After treatment with MT, the decrease in the inflammatory process and cell death, in addition to the presence of granulation tissue are reflected in the measurements of R_L_ and X_H_, which decrease after the regeneration process.

The morphological alterations induced by I/R identified with H-E were accompanied by the loss of acidic glycosaminoglycan-producing cells in the foveolar epithelium (AB-positive) and the reduction of the production of neutral glycosaminoglycans (PAS-positive) in the glandular epithelium. Gastric mucins and mucus layers are secreted from superficial and glandular mucus-producing cells and central peptides are characterized as MUC5AC (synthesized by surface foveolate cells) and MUC6 (synthesized by cells from the neck and glands). After synthesis, mucin accumulates in the form of granules and is then secreted by exocytosis [36, 37]. It has been reported that 75% of critically ill patients present evidence of altered mucus production within 24 hours of admission to the intensive care unit [38]. The loss of mucus-producing cells in the superficial epithelium after stress-induced gastric damage [37] and gastric I/R has been previously reported [39]. Following treatment with MT, the regeneration of AB-positive cells in the foveolar was observed, as well as an increase in the production of neutral glycosaminoglycans, suggesting that the treatment reversed the damage allowing these cell types to maintain barrier function.

The mechanisms of gastric mucosal injury induced by the reintroduction of oxygen into ischemic tissue are associated with the generation of ROS and nitrogen, which lead to the elevation of neutrophil infiltration producing more free radicals and inflammatory mediators, which can lead to lipoperoxidation [40]. In addition, there is an increase in the expression of pro-inflammatory molecules (iNOS and COX-2) which are produced by the activation of NF-κΒ, which initiates the expression of genes involved in inflammatory responses [41–44].

Previous studies have reported that pretreatment with MT produces an inhibitory effect on the expression of iNOS as it inhibits the signaling pathways of MAPK p38 and NF-κΒ, which regulate its expression [42]. This deregulation decreases the production of NO and consequently the creation of peroxynitrite which would limit the damage induced by reperfusion. The results in this work showed that I/R damage induces the expression of iNOS while MT treatment caused apparent negative regulation in its expression. However, after 24 hours of reperfusion, iNOS expression levels could be decreasing, iNOS has been reported to be overexpressed early in reperfusion (1 hour) and then gradually decrease [41].

On the other hand, immunodetection of COX-2 showed an increase in expression mainly in areas of erosion after reperfusion, while decreased with treatment as there was a process of recovery. In gastric mucosa, the increase in the expression of COX-2 and PGE2 has been detected mainly in superficial mucus-producing cells and foveolas adjacent to the ulcerated area [45, 46]. It has been reported that pretreatment with MT reduces the phosphorylation of IκΒ-α, resulting in a reduction in the activation of NF-κΒ, decreasing the expression of COX-2 in models of inflammatory response *in vitro* and *in vivo* [47, 48].

The gastric mucosa has the inherent ability to repair quickly (approximately 24 hours) after a mild and moderate injury by two different repair mechanisms involved in the complete restoration of the injured gastric mucosa; the restitution process, which begins with the migration of viable epithelial cells from the gastric pits and glands, and the proliferation process, which is slower and replaces lost cells in damaged tissue [32]. The proliferating cell nuclear antigen (PCNA) is a protein associated with DNA polymerase δ and is used as a marker of cell proliferation [1]. In *in vitro* models, the application of MT has been shown to have a protective effect by inhibiting the generation of ROS and lipoperoxidation, increasing the rate of proliferation and cell migration, as well as reducing apoptosis in HUVEC cells [49, 50]. In a renal I/R model, Wu *et al*. [51], identified the increase in PCNA-positive cells in the proximal tubular epithelium after I/R, which indicates the initiation of the repair process. In our results, the number of PCNA-positive nuclei tends to increase in the MT-treated group with respect to I/R, although it is not statistically significant. Previously, MT has been reported to increase proliferation and migration, suggesting that it may speed up the process of renewal in the intestinal mucosa [52]. This could indicate that, due to the time of reperfusion, in both experimental groups there is a process of regeneration of the mucosa, however, treatment with MT could accelerate it, which was confirmed with the histological analysis. Proliferative activity has been reported to be time dependent on reperfusion, indicating that the severity of mucosal damage has a negative correlation with proliferative activity [1].

The apoptosis assay TUNEL showed a statistically significant increase in the number of cells in the apoptotic process, which is reversed after treatment with MT. Pretreatment with MT has been shown to have a gastroprotective effect of I/R damage by reducing apoptosis [20]. Similar results have been reported by Wang *et al*. [53] in a lung I/R model. The anti-apoptotic effect induced by MT administration is due to the inhibition of important steps of activation of the mitochondrial pathway of apoptosis such as the overregulation of Bax and Bak expression and deregulation of Bcl-2 and BclxL. MT prevents the mitochondrial translocation of Bax and the collapse of mitochondrial membrane potential and reduces the activation of caspase 3 and 9 by blocking the release of cytochrome C, therefore reducing cell death by an apoptotic route [54].

This study has some limitations, since intermediate I/R groups (3, 6 and 12 hours of reperfusion) were not evaluated, it is not possible to demonstrate the effect of administration of MT on the progression of tissue changes and their relation to the electrical properties of gastric tissue. On the other hand, it would also be relevant to carry out additional studies at the level of MT receptors (MT1 and MT2) to further investigate the mechanism of the effects of MT on changes in electrical impedance.

However, it must be considered that the relevance of this work is to identify that for critically ill patients, in whom the integrity of the gastric mucosa is compromised, as has already been reported [6, 55], melatonin could have positive effects that help prevent the gastric damage. It was possible to show the repair process of the gastric mucosa at 24 h of reperfusion, which were reflected in the changes of the impedance parameters.

Early detection of gastrointestinal ischemic events is particularly relevant in critically ill patients where classic symptoms of abdominal pain are hidden due to sedation and mechanical ventilation. An earlier diagnosis may result in early therapeutic intervention that may prevent the need for surgery or if surgery was needed, it would occur earlier, reducing mortality. Even a 24-hour delay in diagnosis has showed that the survival rate decreases by 20% [4]. In addition to the early identification of gastrointestinal ischemic events in critically ill patients, early therapeutic intervention is important in order to minimize/regenerate induced injury after reperfusion, reducing the different complications that can lead to the patient’s death.

Our group has been working for several years to identify and quantify early gastric mucosal damage through impedance spectroscopy [6, 55], and with this work we evaluate a possible treatment that is sensitive to changes in impedance, a tool that can be used clinically to monitor critically ill patients.

## Conclusion

According to the results the electrical properties od gastric tissue were modified by MT treatment after I/R-induced lesions in 24 hours, which are associated with changes in the analyzed markers of inflammation, proliferation and apoptosis A decrease in impedance parameters (resistance and reactance) was recorded in the treated group with respect to the group without treatment, observing a greater re-epithelization process and less cell death with MT treatment. This suggests that EIS can be used to monitor both damage and recovery of gastric tissue under I/R conditions.

## Supporting information

S1 Data (XLSX)
